# Antimicrobial efficacy of direct air gas soft jet plasma for the in vitro reduction of oral bacterial biofilms

**DOI:** 10.1038/s41598-024-61438-z

**Published:** 2024-05-13

**Authors:** Valentina Puca, Beatrice Marinacci, Morena Pinti, Federica Di Cintio, Bruna Sinjari, Maria Carmela Di Marcantonio, Gabriella Mincione, Tirtha Raj Acharya, Nagendra Kumar Kaushik, Eun Ha Choi, Michele Sallese, Simone Guarnieri, Rossella Grande, Vittoria Perrotti

**Affiliations:** 1 Department of Pharmacy, University “G. d’Annunzio” of Chieti-Pescara, Via dei Vestini 31, 66100 Chieti, Italy; 2 Department of Innovative Technologies in Medicine and Dentistry, University “G. d’Annunzio” of Chieti-Pescara, Chieti, Italy; 3 Department of Oral, Medical and Biotechnological Sciences, University “G. d’Annunzio” of Chieti-Pescara, Chieti, Italy; 4 Plasma Bioscience Research Center, Department of Electrical and Biological Physics, Kwangwoon University, Seoul, 01897 South Korea; 5 Department of Neuroscience, Imaging and Clinical Sciences, University “G. d’Annunzio” of Chieti-Pescara, Chieti, Italy; 6 Center for Advanced Studies and Technology (CAST), University “G. d’Annunzio” of Chieti-Pescara, Chieti, Italy; 7 UdA-TechLab, Research Center, University “G. d’Annunzio” of Chieti-Pescara, 66100 Chieti, Italy

**Keywords:** Bacteria, Biofilms, Dental diseases

## Abstract

The aim of this study was to evaluate the antimicrobial efficacy of an air gas soft jet CAP for its potential use in removing oral biofilms, given that plasma-based technologies have emerged as promising methods in periodontology. Two types of biofilms were developed, one by *Streptococcus mutans* UA 159 bacterial strain and the other by a complex mixture of saliva microorganisms isolated from a patient with periodontitis. This latter biofilm was characterized via Next Generation Sequencing to determine the main bacterial phyla. The CAP source was applied at a distance of 6 mm for different time points. A statistically significant reduction of both CFU count and XTT was already detected after 60 s of CAP treatment. CLSM analysis supported CAP effectiveness in killing the microorganisms inside the biofilm and in reducing the thickness of the biofilm matrix. Cytotoxicity tests demonstrated the possible use of CAP without important side effects towards human gingival fibroblasts cell line. The current study showed that CAP treatment was able to significantly reduce preformed biofilms developed by both *S. mutans* and microorganisms isolated by a saliva sample. Further studies should be conducted on biofilms developed by additional saliva donors to support the potential of this innovative strategy to counteract oral pathogens responsible for periodontal diseases.

## Introduction

The oral cavity is the home of diverse microbial content, named oral microbiome. This can be friend and foe too, and the possible interaction with the human body can be protective and pathogenic^1^. The disruption of normal microflora is known as dysbiosis, this can contribute to various oral diseases such as dental caries or periodontitis^2^. These oral diseases can be correlated to a dental biofilm. Biofilms are complex and dynamic organisations constituted by microorganisms embedded in a self-produced Extracellular Polymeric Substances (EPS) matrix^3^. The biofilm represents a microbial survival strategy in response to stressful stimuli associated with long-term successful infection^4^; in which the planktonic cells reversibly attach to a surface, which is followed by irreversible binding and then multiplication into microcolonies that produce EPS^5^. In particular, oral microbial communities assemble into these complex architectures and their combined actions, shaped by both intra-community interactions and host, and environmental variables, determine homeostatic balance or dysbiotic disease^6,7^. The issues linked to human biofilm infections arise from two separate features shared by all biofilms. The biofilm allows microorganisms to escape the immune system and the antimicrobial drugs attack because of the protection of the EPS matrix^4,8^ that prevent diffusion of antimicrobials into the biofilm. Furthermore, the EPS-associated degradative enzymes, hypermutability, and persisted cells, are just a few examples of how these complex communities survive and adapt to antimicrobial challenge^9^. Polymicrobial biofilms are involved in the Anti-Microbial Resistance (AMR) phenomenon. Unfortunately, the misuse and/or the abuse of antimicrobials in animal and human medicine have accelerated the growing worldwide phenomenon of AMR^4^. Superbugs and multidrug-resistant bacteria are endemic in many parts of the world and the presence of inhibitory and sub-inhibitory concentrations of antimicrobials in natural habitats could bring out novel resistance mechanisms. For these reasons, the AMR results in the top ten global public health threats facing humanity^10,11^. In terms of the global burden of increasing antibiotic resistance development, restricted use of antimicrobials is suggested in the clinical treatment of the oral cavity diseases^12^. The detection of new antimicrobial and particularly antibiofilm strategies represents an important approach to prevent AMR. In this scenario, Cold Atmospheric Plasma (CAP) could be a promising alternative strategy to counteract microbial infections related to biofilms, in addition, the mechanism of action of plasma is different from that of an antimicrobial agent.

Recently, CAP has emerged as a new therapy that can deliver Reactive Oxygen Species (ROS) and Reactive Nitrogen Species (RNS), for biomedical applications^13–20^. CAP is a multi-component, chemically active, and highly reactive ionized gas that is generated at room temperature under atmospheric conditions, usually from noble gases (i.e., helium or argon), and flows into ambient air or is directly created in air. The species created by CAP are mainly RNS, such as NO• and nitrogen dioxide (NO_2_), as well as ROS, such as ozone (O_3_), OH•, O_2_^−^, ^1^O_2_, and H_2_O_2_^14,16,21,22^. All these reactive species can reduce the bacteria viability.

CAP has shown to induce a variety of biological effects, such as blood coagulation^23^, tissue regeneration^24,25^, sterilization^13,23,26^, wound healing^23,27,28^, cancer cell death^14–17,21,23,26,29,30^, activation of immune cells^31,32^, and virus inactivation^33,34^. The type and concentration of CAP-generated species delivered to cells depend on the CAP operating conditions, controlled by the design of the source, including the configuration of the electrodes. The clinical and experimental uses of CAP can include two different modalities: the direct and indirect applications. Direct treatment involves the direct exposure of the biological target to plasma in the presence of a liquid or not, while indirect treatment involves the treatment of a liquid, and the application of this plasma-activated solution onto the biological target^35^.

The direct application of CAP to human tissues is approved by the European Committee for Standardization (CEN) and the International Organization for Standardization (ISO) for wound healing and head and neck cancer^36^. However, the direct application of CAP has some limitations due to the limited feasibility of delivering ROS and RNS to internal target tissues.

In literature, numerous CAP inhibition mechanisms have been described based on the classification of cellular structural components, including oxidation and perforation of cell membrane, degradation and modifications of proteins, modification and chain-breaking of nucleic acid molecules, and disruption of EPS and interference with Quorum Sensing (QS) on biofilms^37,38^.

The aim of this study was to evaluate the antimicrobial efficacy of an air gas soft jet CAP for its potential use in removing oral biofilms.

## Results

### Physical and chemical characterization of air gas soft jet plasma device

**Figure 1:**
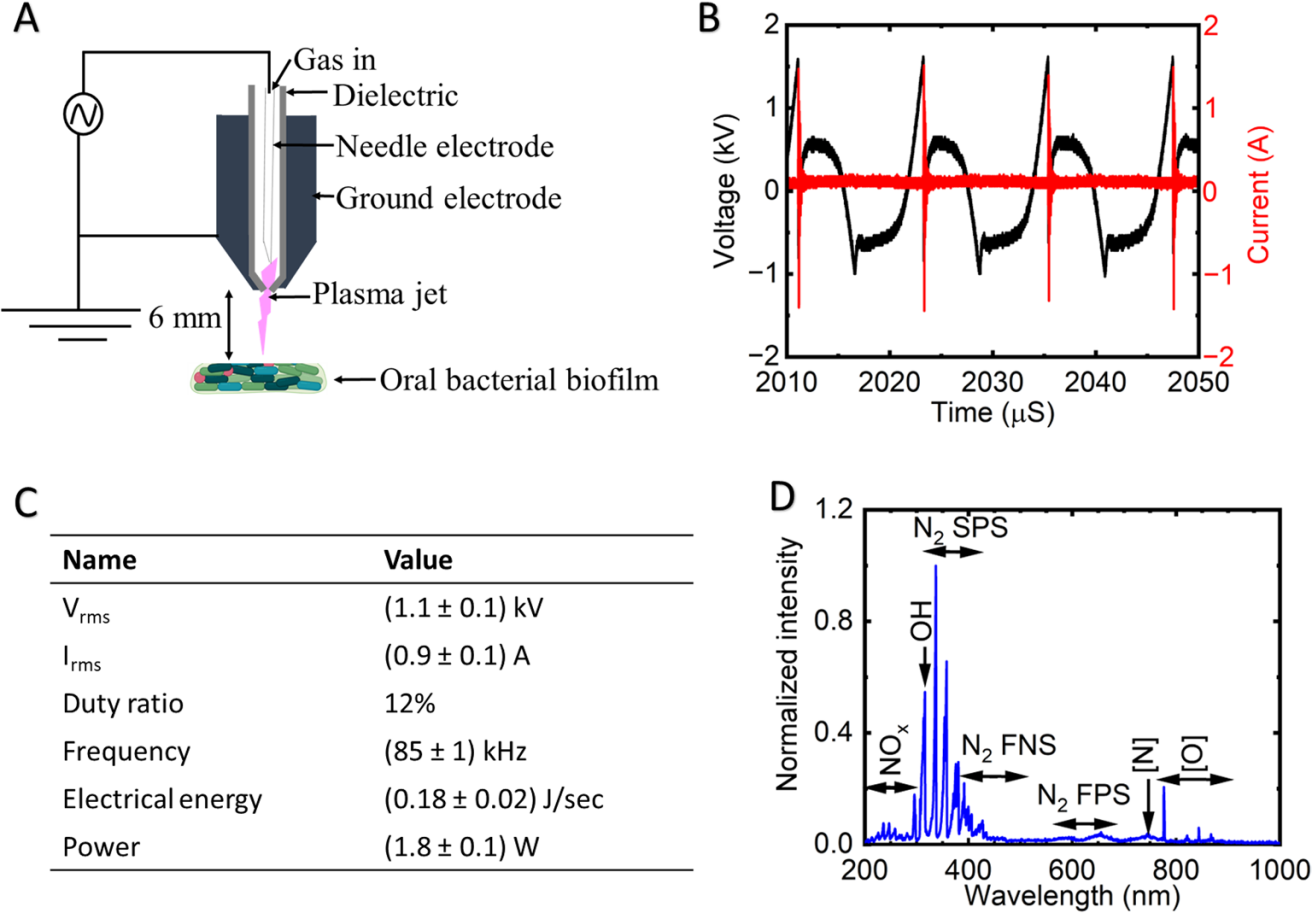
Illustration depicting the configuration of a soft jet plasma (**A**), current–voltage characterization of the soft jet plasma (**B**), electrical properties of the soft plasma jet (**C**), optical emission spectra of the soft plasma jet (**D**).

Figure 1B illustrates the discharge waveforms generated using air, showcasing the characteristic patterns of current and voltage. In Fig. 1C, the current–voltage characterization of the plasma is presented, providing the numerical values used for plasma diagnostics. The peak current, calculated using the root mean square (rms) method, is approximately 0.9 A, while the peak voltage, also determined through rms, reaches around 1.1 kV. The voltage is regulated by employing a duty ratio of 12% with 31 ms of on time. The frequency, electrical energy and power of the plasma were 85 kHz, 0.18 J/s and 1.8 W, respectively. The plasma generated energy, and dissipated power were calculated using the following equations^39,40^:

1 $$\mathrm{Energy }\left({\text{E}}\right)=\mathrm{ Q}\times \mathrm{V }= {\int }_{{\text{T}}1}^{{\text{T}}2}{\text{V}}\left({\text{t}}\right){\text{I}}\left({\text{t}}\right){\text{dt}} [{\text{J}}]$$

2 $$\mathrm{Power }({\text{P}}) =\mathrm{ Duty\, ratio }\times \frac{1}{{\text{T}}}{\int }_{0}^{{\text{T}}}{\text{V}}\left({\text{t}}\right){\text{I}}\left({\text{t}}\right){\text{dt}}[{\text{W}}]$$

Here, the duty ratio can be defined as:

3 $${\text{Duty ratio}} = \frac{{\text{On time}}}{{\left( {{\text{On time}} + {\text{Off time}}} \right)}} \times 100 [\% ]$$

where, Q = Charge, V = Peak voltage, I = Peak current, T = Plasma discharge time.

Furthermore, Fig. 1D presents the Optical Emission Spectra (OES) of the soft jet plasma in the range of 200–1000 nm conducted in this experiment. The NO_X_ spectrum was examined within the wavelength range of 200–280 nm, revealing the presence of OH radicals emitting at 309 nm. The emission attributed to the nitrogen second positive (N_2_ SPS) was observed in the 311–380 nm region^40^. Furthermore, within the range of 390–410 nm, the First Negative System (FNS) of nitrogen (N_2_) emission was observed. Also, between 550 and 700 nm, the First Positive System (FPS) was observed. Notably, atomic nitrogen (N) exhibited a distinct light emission at 780 nm, while atomic oxygen (O) emission was observed between 777 and 845 nm^39^. The electron temperature (kTe) was measured to be 1.15 eV, while the electron density (N_e_) was determined to be 2.80 × 10^14^ cm^-3^*.* The densities of the nitrogen meta-stable states (N_A_, N_B_, and N_C_) were found to be 2.73 × 10^15^ cm^−3^, 2.04 × 10^15^ cm^−3^, and 1.28 × 10^14^ cm^−3^, respectively. Additionally, the vibrational temperature (T_v_) and rotational temperature (T_g_) of the cold soft jet plasma were calculated to be 0.65 eV and 730 K, respectively.

**Figure 2:**
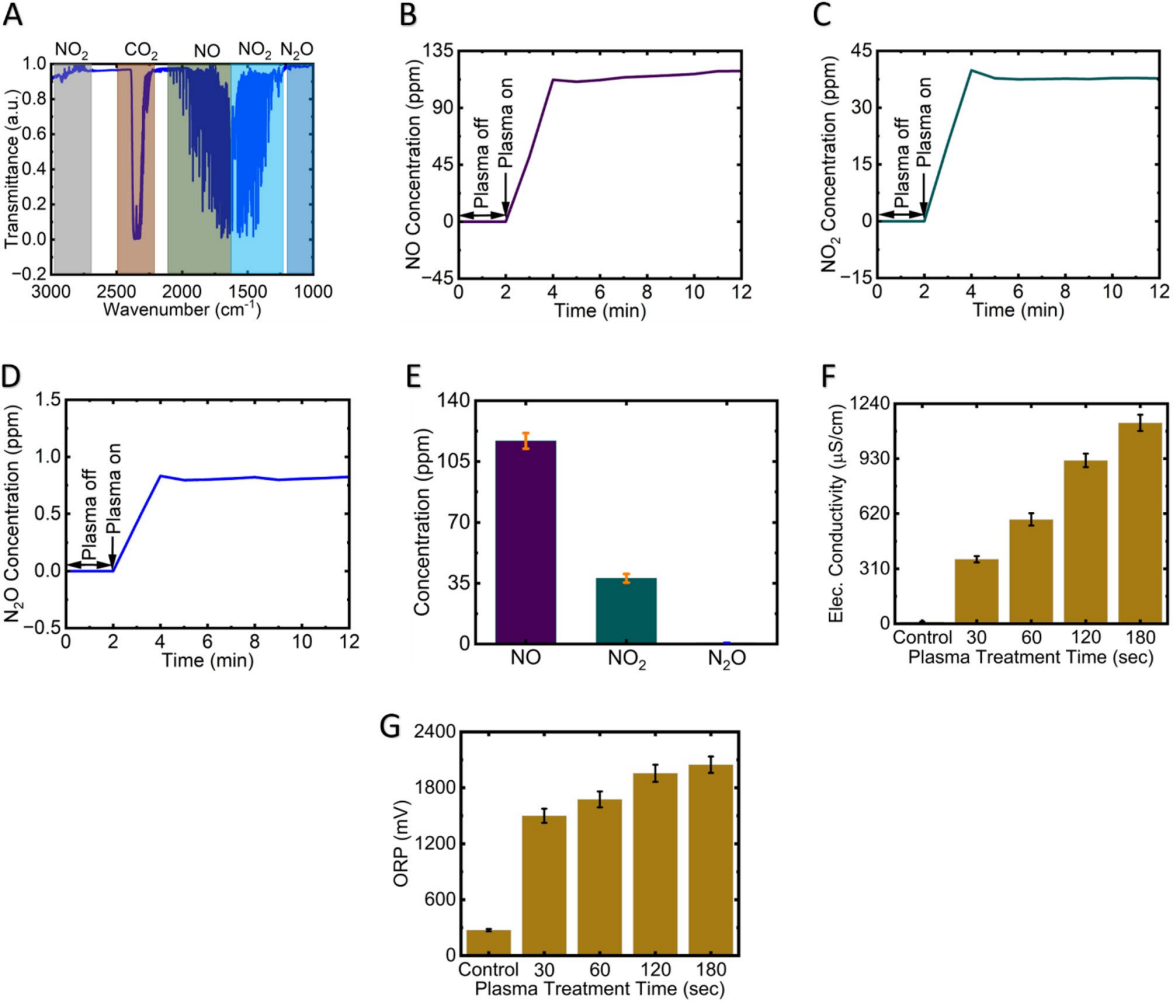
Fourier Transform Infrared (FTIR) transmittance spectrum of soft jet plasma in the wavenumber range of 3000–100 cm^−1^ (**A**), time-dependent concentration profiles of reactive species NO (**B**), NO_2_ (**C**), N_2_O (**D**), average concentration profiles of reactive species after 10 m of soft jet plasma treatment (**E**), electrical conductivity (**F**), and Oxidation Reduction Potential (ORP) of plasma treated deionized water (**G**).

Figure 2A displays the FTIR spectroscopy analysis conducted to determine the gas phase composition of the plasma. The FTIR spectra captured within the range of 3000–1000 cm^−1^ revealed the presence of various plasma-generated gases, including NO (1700–2000 cm^−1^), NO_2_ (1540–1660 cm^−1^ and 2840–2940 cm^−1^), and N_2_O (1250–1350 cm^−1^). Figure 2B, C, D and E illustrate the variations in the concentrations of plasma-generated NO, NO_2_, and N_2_O before and after plasma treatment over a time span of 0–12 m. Notably, no reactive species were observed during the initial 0–2 m period, indicating their absence prior to plasma generation. However, following the initiation of plasma synthesis 2–12 m, distinct reactive species, particularly NO, NO_2_, and N_2_O, became evident, indicating their formation during this specific timeframe. Notably, NO exhibited a dominant concentration, with an average concentration of approximately 116.9 ppm over a 10 min of plasma treatment period. In contrast, NO_2_ and N_2_O demonstrated lower average concentrations of approximately 37.9 ppm and 0.7 ppm, respectively, over the same 10 min plasma exposure time. It is important to mention that other plasma-generated gases such as HNO_3_ and O_3_ were not detected in this analysis. Figure 2F depicts the electrical conductivity of Deionized Water (DW) before and after plasma treatment for durations of 30, 60, 120, and 180 s. The initial electrical conductivity of DW before plasma treatment was 10μS/cm, while after 30 s, 60 s, 120 s, and 180 s of plasma treatment, the electrical conductivity increased to 364, 588, 920, and 1132 μS/cm, respectively. In Fig. 2G, the changes in electrical conductivity of deionized water (DW) before and after plasma treatment are illustrated for durations of 30 s, 60 s, 120 s, and 180 s. Initially, the ORP of DW before plasma treatment measured 274 mV. However, following plasma treatment for 30 s, 60 s, 120 s, and 180 s, the electrical conductivity increased to 1500, 1676, 1956, and 2048 mV, respectively. The increase in electrical conductivity and ORP of plasma-treated deionized water (DW) with plasma exposure time can be attributed to several factors. Plasma treatment generates reactive species like ions and free radicals that interact with water molecules, increasing ionization. This elevated ion presence contributes to higher electrical conductivity. Dissociation of water molecules into hydrogen ions (H^+^) and hydroxide ions (OH^−^) due to plasma treatment further enhances electrical conductivity. Plasma-induced oxidation and reduction reactions modify the redox potential, resulting in an increased ORP. The generation of reactive oxygen and nitrogen species during plasma treatment also contributes to the rise in ORP.

### Evaluation of saliva biofilm formation after 24, 48 and 72 h of incubation

The biofilm formed by the microorganisms isolated from a saliva sample at different time points has been studied, in order to establish the best time of incubation before CAP treatment. A biofilm with a huge number of CFU was already formed after 24 h of incubation, as shown in the XTT assay and CFU count graphs (Supplementary Figure S1A–B). After 48 and 72 h of incubation the biofilm biomass increased (Supplementary Figure S1C) while the cellular viability remained almost stable (Supplementary Figure S1A, B).

### Next generation sequencing of bacteria

Planktonic cells derived by the overnight broth culture (plank ON) and sessile cells derived from the biofilm developed after 24, 48, and 72 h of incubation, in triplicate, were subjected to next generation sequencing (NGS). Sequencing generated a total of 1,164,904 reads, equivalent to 9,7075 means of reads per sample (range 7,3145–123,629).

### Bacterial community profiling

**Figure 3:**
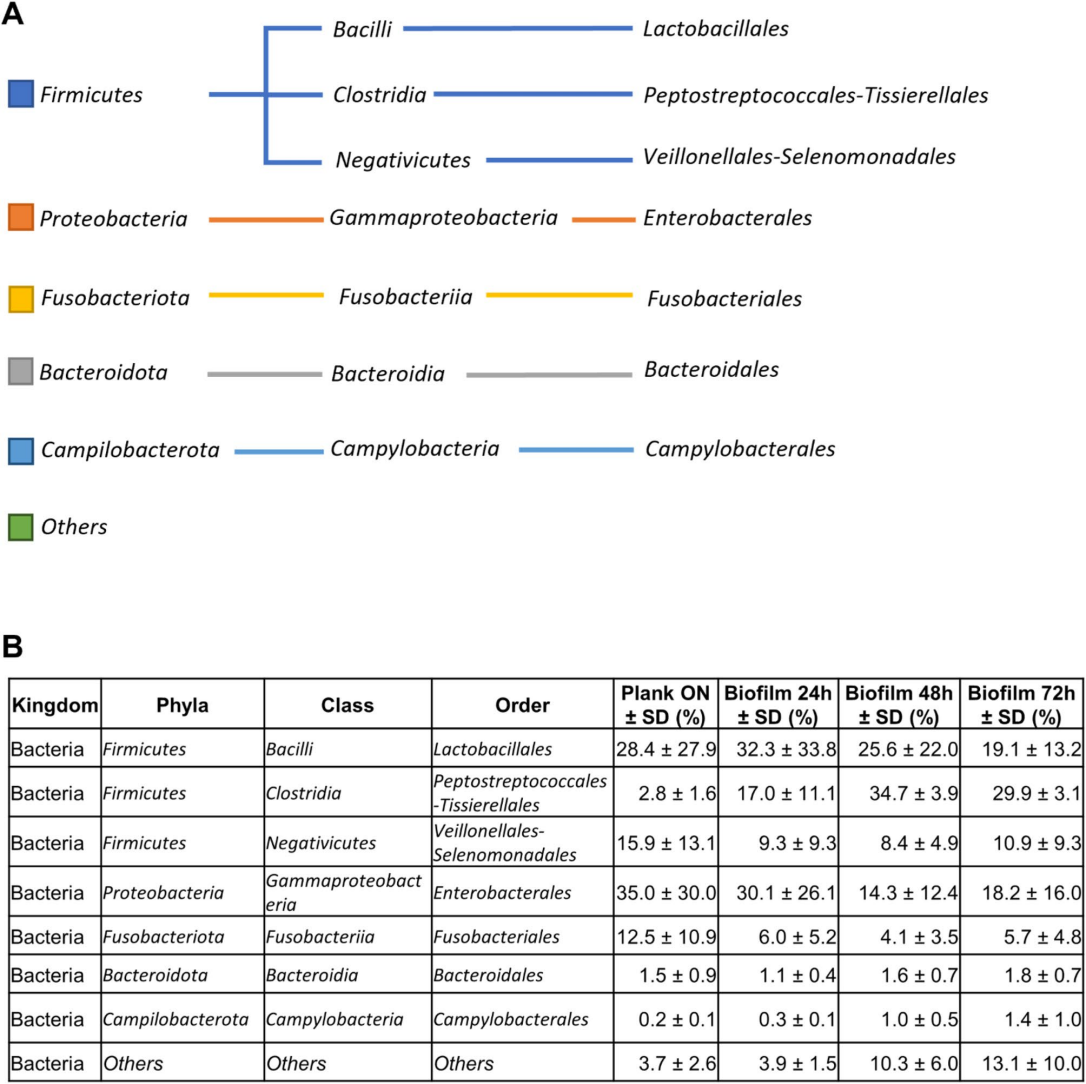
Comparative taxonomic analysis of the overnight planktonic cells (plank ON) and biofilm after 24, 48,72 h of formation. Panel (**A**) Representative scheme of taxonomic classification obtained from 16S rDNA sequencing of the plank ON, biofilm developed at 24, 48, and 72 h of incubation. Panel (**B**) Table of microbial diversity is inferred from 16S rDNA taxonomic assignment, from reads obtained by sequencing the 16S rDNA of the plank ON, biofilm at 24, 48, and 72 h of formation. The *phylum*-level taxonomic structure revealed marked differences in the community profiles between the plank ON and biofilm after 24, 48, and 72 h. The top 5 *phyla* in terms of maximum relative abundance are shown. All other *phyla* with low or no presence (≤ 0.7) were grouped as “Others”. The *phyla* were subdivided into the members of the class and order. Data are expressed as means ± SD (n = 3).

Plank ON and biofilm cells were analysed for taxonomic study by 16S ribosomal DNA sequencing. The bacteria identified belonged to 7 different *phyla* and 50 different *genera*. *Phyla* that were very poorly represented were not further considered (≤ 0.7%). The five most represented *phyla* were, in descending order of quantity: *Firmicutes*, *Proteobacteria, Fusobacteriota, Bacteroidota,* and *Campilobacterota* (Fig. 3A, B and Supplementary Figure S2).

The planktonic cells were represented by *Firmicutes* (49.7% ± 40.1), *Proteobacteria* (35.0% ± 30.2), *Fusobacteriota* (6.5% ± 11.4), *Bacteroidota* (1.5% ± 0.9), and *Campilobacterota* (0.2% ± 0.1) (Supplementary Figure S2). Within the *Firmicutes* phyla the classes of *Bacilli, Clostridia* and *Negativicutes* and the order of *Lactobacillales*, *Peptostreptococcales-Tissierellales* and *Veillonellales-Selenomonadales* were identified. Within the *Proteobacteria* the classes of *Gammaproteobacteria* and the order of *Enterobacterales* were identified. Within the *Fusobacteriota* the class of *Fusobacteriia* and the order of *Fusobacteriales* were identified. Within the *Bacteroidota* the class of *Bacteroidia* and the order of *Bacteroidales* were identified*.* Finally, within the *Campilobacterota* the class of *Campylobacteria* and the order of *Campylobacterales* were identified (Fig. 3B).

Comparing the planktonic phenotype composition with that of the biofilm phenotype it was evident that *Firmicutes* and *Campilobacterota* increased (*p* < 0.05) while *Proteobacteria* and *Fusobacteriota* decreased over time (Fig. 3B and Supplementary Figure S2). Finally, *Bacteroidota* remained rather constant in the biofilm phenotype as compared to plank ON phenotype.

**Figure 4:**
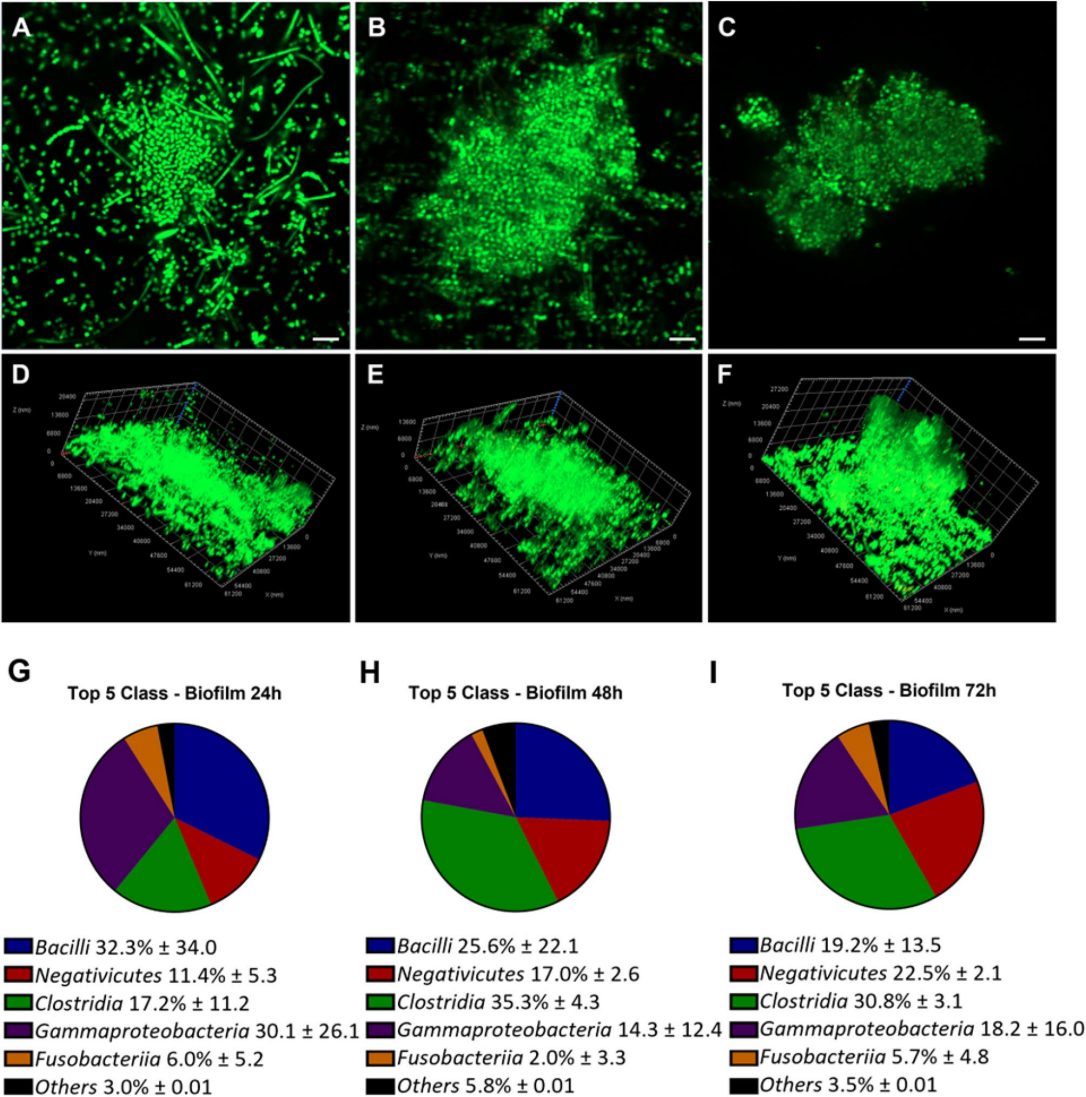
Microbial community structure in the biofilms formed after 24, 48, and 72 h. Representative images of biofilm developed from microorganisms isolated from the saliva sample after 24 h (**A**, **D**), 48 h (**B**, **E**) and 72 h (**C**, **F**) of incubation via live/dead staining and CLSM (represented as Z projection and orthogonal projection). Bar: 5 µm. Below each image, the class composition of microorganisms obtained by 16SrDNA sequencing in biofilm samples after 24 h (**G**), 48 h (**H**), and 72 h (**I**).

A similar comparison at the class level, revealed a high presence of *Bacilli*, *Gammaproteobacteria, Negativicutes and Fusobacteriia*. In the biofilm cultured for 24 h, the amounts of *Gammaproteobacteria* and *Negativicutes* were reduced in favour of *Clostridia* and *Fusobacteriia*. (Fig. 4).

After 48 h, the *Bacilli* and *Gammaproteobacteria* in the biofilm sample showed a decreasing trend. *Clostridia* showed a significant increase (*p*-value = 0.0002); *Negativicutes* also increased, although not significantly while *Fusobacteriia* remained unchanged.

After 72 h, the *Clostridia* showed a significant increase (*p*-value = 0.0001) in the biofilm phenotype compared to the planktonic one. The *Negativicutes* seem to increase too, but not significantly. The *Fusobacteriia* still remained unchanged (Fig. 4).

A deeper investigation, which considered the bacterial orders (Fig. 3B), indicated that *Lactobacillales* in the biofilm samples were similar after 24 and 48 h and tended to decrease at 72 h. The *Peptostreptococcales-Tissierellales* significantly increased both after 48 and 72 h (*p*-value = 0.0002) while the *Campylobacterales* significantly increased only after 48 h (*p*-value = 0.05). The *Veillonellales-Selenomonadales* and *Bacteroidales* remained almost unchanged in all biofilm samples. Finally, the relative amount of *Enterobacterales* and *Fusobacteriales* showed a decreasing trend.

The Confocal Laser Scanning Microscopy (CLSM) analysis was used to support the data obtained via the CFU count, XTT and CV assay and to observe the bacterial morphologies in the saliva biofilms. After 24 h of incubation the biofilm appeared with numerous cells adhering to the bottom of the plate, most of them with a coccoid morphology and some aggregates (Fig. 4A and D). The aggregates were formed by multi-species bacteria, as demonstrated by the different morphologies observed in the samples: cocco-bacillary, coccoid and only a few fusiform cells. The thickness of the biofilm aggregates developed after 24 h of incubation was in the range of 10–22 µm. After 48 h of incubation the biofilm appeared very variable when observing different areas of the Petri dish. There were aggregates of coccoid cells, as observed in the 24 h biofilm; however, monolayers of bacillary cells adhered to the bottom of the plate were also observed. The 48 h biofilm aggregates showed greater size and more complex structure than the 24 h biofilm (Fig. 4B and E). The thickness of the biofilm aggregates developed after 48 h of incubation was similar to that of the 24 h biofilm. The biofilm developed after 72 h of incubation was characterized by carpets of bacillary and fusiform cells at the bottom of the Petri dish and above them, mushrooms of bacterial cells were rising from the bottom to the top of the dish. The aggregates were larger and more organized (Fig. 4C and F) than the aggregates observed in the 48 h biofilm. The thickness of the biofilm aggregates developed after 72 h of incubation was grater and was in the range of 10–30 µm. In consideration of the fact that the microbial composition of the biofilm remained rather constant over time, the biofilm developed after 72 h of incubation was used for the subsequent experiments because it demonstrated to be more complex and well structured.

### Determination of CAP minimum biofilm eradication concentration (MBEC) versus the biofilms developed by S. mutans UA 159 and microorganisms isolated from the saliva sample

**Figure 5:**
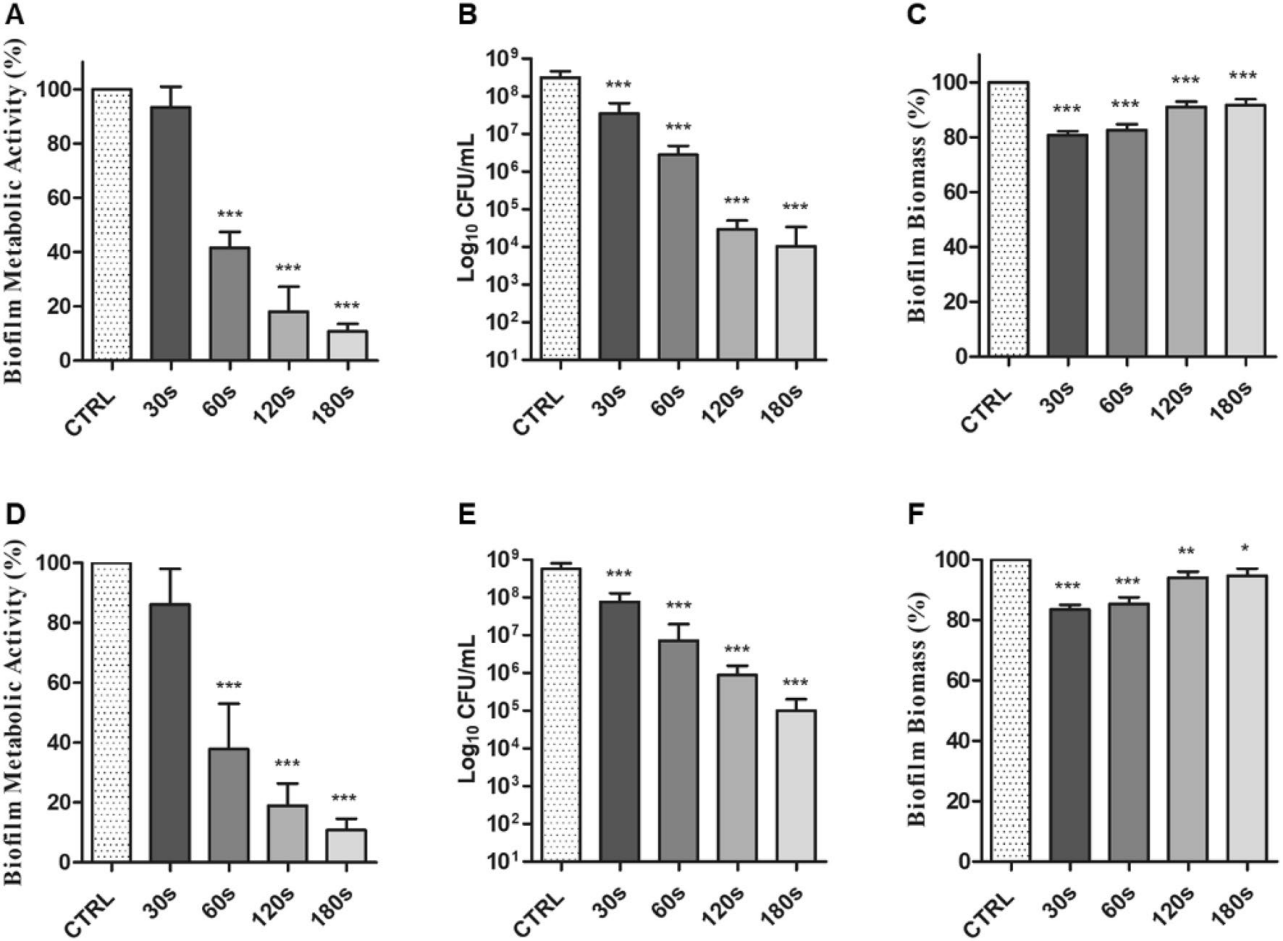
In vitro activity of CAP against *S. mutans* UA 159 preformed biofilm (**A**, **B**, **C**) and against the preformed biofilm developed by microorganisms isolated from a saliva sample (**D**, **E**, **F**). CTRL: control, untreated biofilm; 30 s, CAP treatment for 30 s; 60 s, 60 s; 120 s, 120 s; 180 s, 180 s. Histograms represent *S. mutans* biofilm reduction after CAP treatment expressed as the percentage of the biofilm metabolic activity (**A**); the CFU count (**B**); the percentage of the biofilm biomass measured via CV staining (**C**). XTT and CV results are normalized to the untreated biofilm. The results represent the mean ± SD of three independent experiments. *p < 0.05 vs the control (**C**), **p < 0.005 vs C; ***p < 0.001 vs C. (ANOVA + Dunnett’s Multiple comparison test).

The first step was the determination of the effectiveness of CAP *versus Streptococcus mutans* 24 h biofilm via the determination of the MBEC. The MBEC was defined as the lowest concentration of an antimicrobial substance that eradicates 99.9% of biofilm-embedded bacteria (which corresponds to 3 log10 reduction in CFU/mL) compared to growth controls^41,42^. CAP treatment was effective in reducing the biofilm developed by *S. mutans* UA 159. A statistically significant reduction of the CFU, with a *p*-value less than 0.001 vs the control biofilm, was already detected after 60 s of CAP treatment while biofilm reduction related to a statistically significant decrease of − 4Log_10_CFU/mL was reached after 120 s of treatment, as shown in Fig. 5B. In accordance with the CFU counts the metabolic activity decreased by of 60% after 60 s and by about 80% after 120 s of CAP (Fig. 5A). Regarding the evaluation of the biofilm biomass treated with CAP, different results were obtained: the CV stained the biomass of the biofilm treated at each time points, with a significant, although slight, reduction after 30 s and 60 s of CAP treatment and without differences when comparing 120 s and 180 s CAP treatment to the untreated biofilm (Fig. 5C).

Subsequently, CAP effect *versus* the 72 h biofilm developed by the microorganisms isolated by the saliva sample was evaluated, obtaining similar results. In detail, CAP was effective in reducing the saliva biofilm after 60 s of treatment, as demonstrated by a statistically significant reduction of both metabolic activity and CFU count (Fig. 5D–F). CAP was slightly less effective in reducing the CFU count when comparing its effect against *S. mutans* biofilm, since a significant reduction of the biofilm was reached after 180 s of treatment, where the CFU reduction was of − 3Log_10_CFU/mL (Fig. 5E). The metabolic activity of the biofilm decreased in dependence of the CAP application time. There was a reduction by about 60% after 60 s of CAP and by 80% after 120 s of CAP (Fig. 5D). As demonstrated for *S. mutans* biofilm, CAP treatment provoked a slight reduction of the biofilm biomass after 30 s and 60 s, but no difference was detected when comparing the results of CAP treatment for 120 s and 180 s (Fig. 5F) with the untreated biofilm. Nevertheless, a statistically significant difference between treated and untreated biofilms was detected.

### CLSM observations of the biofilms treated with CAP

**Figure 6:**
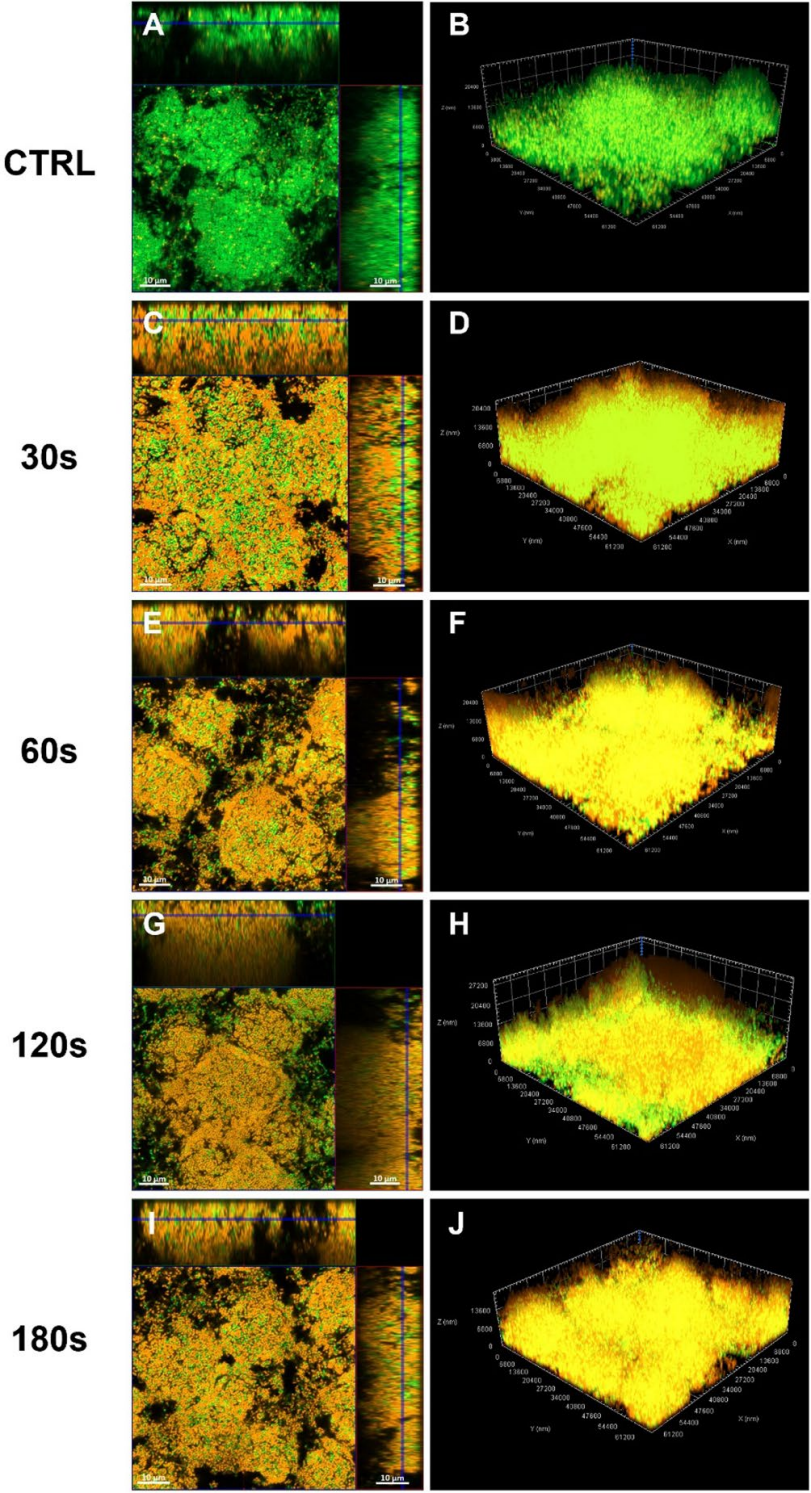
Representative images of *S. mutans* treated biofilm stained with live/dead kit and analysed via CLSM; orthogonal projection and Z projection are reported. untreated biofilm (**A**, **B**); biofilm treated with CAP for 30 s (**C**, **D**); 60 s (**E**, **F**); 120 s (G, H); 180 s (**I**, **J**). Scale bars: 10 µm.

Both *S. mutans* and the saliva biofilms treated with CAP were also analyzed via the CLSM to both confirm the data obtained by using the methods mentioned previously and better understand the effects of plasma treatment. The qualitative analysis by CLSM showed that *S. mutans* untreated biofilm was composed by spheric aggregates which cover the bottom of the Petri dish. The cells had a coccoid morphology and most of them were alive, as demonstrated by the green fluorescence (Fig. 6A). The biofilm was highly structured, and the thickness had an average value of 23–25 µm (Fig. 6A–B). *Streptococcus mutans* biofilm treated with CAP for 30 s seemed to be compressed towards the bottom of the Petri dish. The cells were yellow–orange, indicating that they are dead or are dying. Some alive cells were present inside the biofilm (Fig. 6C). The thickness had an average value of 22–25 µm (Fig. 6C–D). *Streptococcus mutans* biofilm treated with CAP for 60 s was very similar to the biofilm treated for 30 s, but an increased number of dead cells was present (Fig. 6E–F). *Streptococcus mutans* biofilm treated with CAP for 120 s and 180 s were similar with each other when observed at the CLSM. At a glance both Petri dishes appeared to be red when using the Propidium iodide filter, meaning that almost all the cells where dead. *Streptococcus mutans* biofilm treated with CAP for 120 s maintained its 3D structure with a fairly high thickness (Fig. 6G–H). *Streptococcus mutans* biofilm treated with CAP for 180 s presented many more dead cells and the aggregates also had a lower thickness than the biofilms treated at the other time points (Fig. 6I–J).

**Figure 7:**
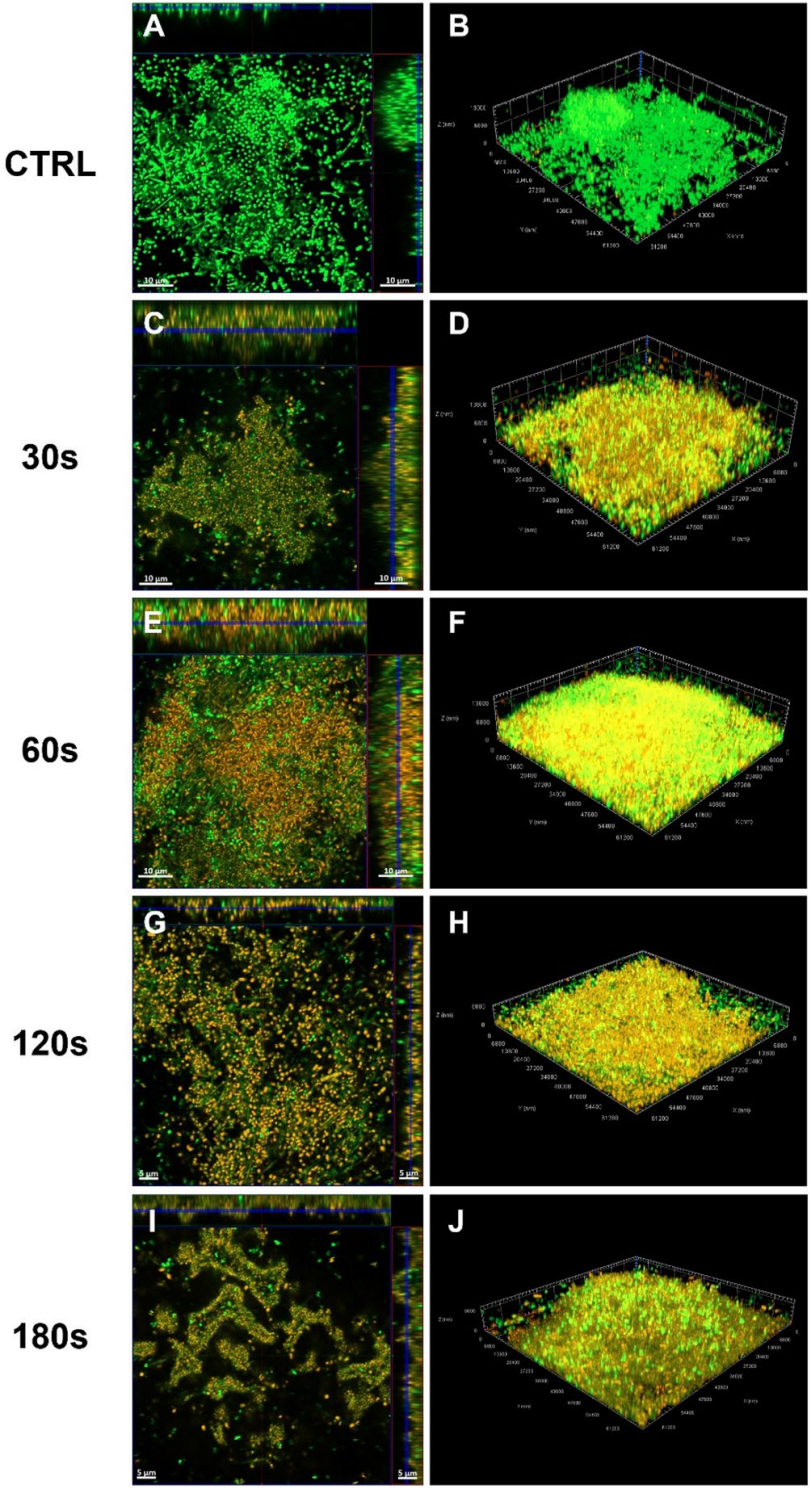
Representative images of saliva treated biofilm stained with live/dead kit and analysed via CLSM; orthogonal projection and Z projection are reported. untreated biofilm (**A**, **B**); biofilm treated with CAP for 30 s (**C**, **D**); 60 s (**E**, **F**); 120 s (**G**, **H**); 180 s (**I**, **J**). Scale bars: (**A**, **C**, **E**) 10 µm; (**G**, **I**) 5 µm.

Subsequently CAP was applied to 72 h biofilm developed by the microorganisms isolated from the saliva sample collected by a patient with periodontitis, in order to establish its efficacy against a multispecies oral biofilm. The untreated biofilm was characterized by carpets of viable cells characterized by many morphologies (Fig. 7A–B). Some mushrooms were rising from the bottom to the top of the Petri dish, and many aggregates with a thickness of 10–30 µm were present, as described above. The aggregates in the saliva biofilm, treated with CAP for 30 s and 60 s were very close together, almost as if they were crushed to each other and compressed towards the bottom of the Petri dish, similar to *S. mutans* biofilm. Both alive and dead cells were present inside the biofilm and many morphologies were observed (bacillary, cocco-bacillary and fusiform cells; Fig. 7C–F). The thickness of the treated biofilm is reduced in respect to the untreated biofilm, in detail, after 60 s of treatment the thickness lowered until 8–15 µm. After the treatment with CAP for 120 s, the biofilm was full of dead cells and at the center of the Petri dish the biofilm was nearly completely detached. Few aggregates were present, the mushrooms were not visible, and the biofilm thickness was very lowered, on the order of 5–7 µm (Fig. 7G–H). The saliva biofilm treated with CAP for 180 s lost its 3D structure, and presented a few cells adhered at the bottom of the Petri dish, almost all of which were dead. The biofilm presented numerous empty channels on the apical surface and the biofilm thickness was similar to that of the biofilm treated after 120 s (Fig. 7I–J).

### Cell viability in response to direct treatment

**Figure 8:**
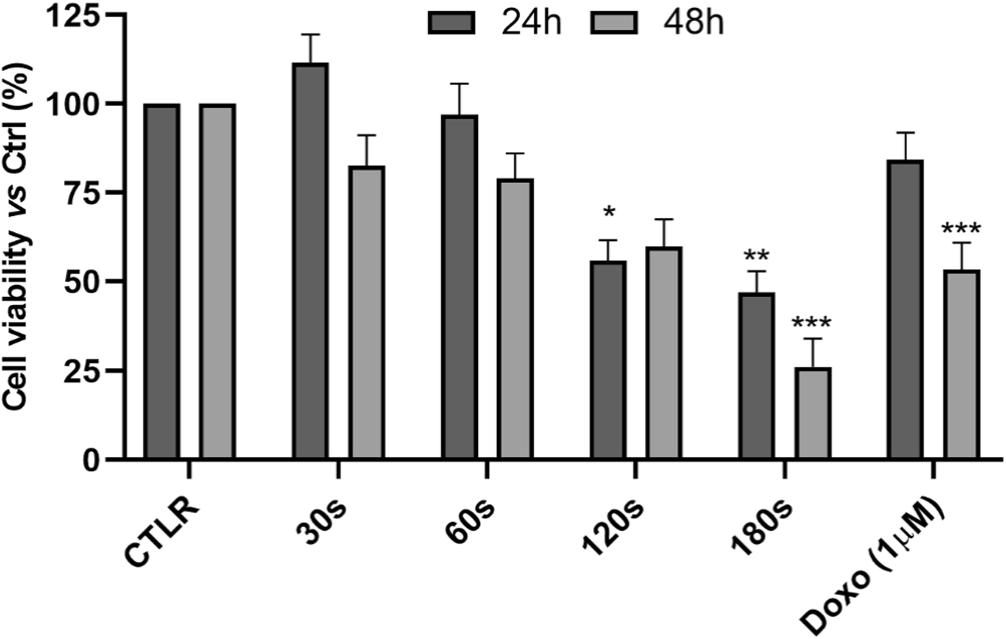
The influence of direct CAP treatment time response on cell viability was evaluated after 30 s, 60 s, 120 s and 180 s of plasma treatment. CTRL: control, untreated cells; 30 s, CAP treatment for 30 s; 60 s, 60 s; 120 s, 120 s seconds; 180 s, 180 s. Cell viability normalized to control cells. The doxorubicin was used as positive control. Data are means ± standard deviation of three independent experiments, each in quintuplicate. *p < 0.05; **p < 0.01; ***p < 0.001 (student’s t-tests). CTRL: control cells; Doxo: doxorubicin.

The effects of CAP direct treatment on hGF cells were assessed. Cell viability was evaluated by MTS assay at 24 h and 48 h after the CAP treatment at 30 s, 60 s, 120 s and 180 s. The working distance between the capillary of the plasma device and the liquid medium surface was fixed at 6 mm. These results (Fig. 8) showed that CAP was able to reduce cell viability, in a time-dependent manner, reaching a peak of reduction at 180 s by ~ 50% after 24 h and by ~ 75% after 48 h compared to untreated cells.

### Effects of CAP direct treatments on hGF cell morphology

To understand whether the CAP direct treatment could have a functional impact on cell morphology, a staining with Toluidine Blue solution on hGF cells was performed. As shown in Supplementary Figure S3, the control cells exhibited typical thin and elongated morphology, while hGF cells stimulated with CAP, in particular at the longer exposure times (120 s and 180 s), showed morphological modifications with loss of their elongated aspect and a reduction in cell number.

## Discussion

Soft jet plasmas are created by applying electrical energy to ambient air, creating a high-energy environment that facilitates unique reactions forming various ROS and RNS^43,44^. The formation of nitrogen oxides (NO_x_), including NO (nitric oxide), NO_2_ (nitrogen dioxide), and N_2_O (nitrous oxide), in a soft jet plasma involves complex chemical reactions^13^. The main reactions leading to NO formation involve the dissociation of nitrogen molecules (N_2_) and their combination with oxygen molecules (O_2_) driven by high-energy electrons (e^−^) in the plasma (Eq. 4). NO_2_ is formed through the oxidation of NO by oxygen (Eq. 5). N_2_O is produced by combining NO and NO_2_ through a condensation reaction (Eq. 6). These NO_x_ species generated by air CAP possess potent antibacterial properties (Eqs. 6 and 7). Bacterial cell death can ensue as NO and NO_2_ directly interfere with and damage bacterial cell membranes and DNA. N_2_O can enhance these effects, further boosting their antibacterial efficacy. Moreover, NO_x_ has the ability to hinder the formation of new biofilms and undermine the structural stability of existing ones.

4 $${\text{N}}_{{2}} + {\text{ e}}^{ - } \, \to {\text{ 2N }} + {\text{ e}}^{ - } \left( {\text{nitrogen dissociation}} \right)$$

5 $${\text{N }} + {\text{ O}}_{{2}} \to {\text{ NO }} + {\text{ O }}\left( {\text{formation of nitric oxide}} \right)$$

6 $${\text{2NO }} + {\text{ O}}_{{2}} \to {\text{ 2NO}}_{{2}} \left( {\text{formation of nitrogen dioxide}} \right)$$

7 $${\text{2NO }} + {\text{ N}}_{{2}} {\text{O}}_{{2}} \to {\text{ 2N}}_{{2}} {\text{O }} + {\text{ O}}_{{2}} \left( {\text{formation of nitrous oxide}} \right)$$

The rising electrical conductivity and ORP in liquids treated with air CAP can be attributed to the ionization of molecules and the generation of ROS and RNS^45^. As the air CAP treatment progresses, these processes lead to the production of ions and oxidative species, resulting in increased electrical conductivity and the establishment of a more oxidative environment with a higher ORP. The extended exposure time during plasma treatment allows for the cumulative effects of these processes, making it valuable for a range of applications, including the removal and control of oral bacterial biofilms.

Periodontal diseases are chronic inflammatory conditions that affect the tissues surrounding and supporting the teeth. Periodontitis is a multifactorial disease driven by the dynamic interactions between oral polymicrobial biofilms and the host immune response and is further affected by other local, environmental, and genetic factors^6,7^.

The results of this study showed that the bacterial composition of the biofilms derived from the microorganisms in the saliva sample of a patient affected by periodontitis, was reproducible by replicates of samples derived from three independent experiments. The obtained biofilms were highly complex and included microbial phyla that commonly colonized the oral cavity. A highly diverse community of bacteria was maintained which included the phyla known to be predominant in the oral cavity such as *Firmicutes*, *Proteobacteria*, *Fusobacteria*, *Bacteroidetes* except for *Actinobacteria* and Spirochaetes^46–48^.

Little is known about the succession of stages leading from periodontal health to disease, and as a result, the unambiguous assignment of bacterial species to periodontal health or periodontitis has proven difficult from the moment that they often show equal prevalence and relative abundance in both health and disease states. Moreover, it is important to note that in vivo studies show substantial variation in the microbiomes among individuals with periodontitis and even between sites in the same individual^6^.

Nevertheless, the relative abundances of microbial species would be expected to change over time as the biofilms developed. Bacteria belonging to the order of *Lactobacillales* and *Enterobacterales* decreased over time whereas bacteria belonging to *Peptostreptococcales-Tissierellales* and *Campylobacterales* increased in the biofilm formed after 48 h of incubation compared with that of 24 h.

Although dental biofilms are complex ecosystem consisting of diverse microbial communities, *S. mutans* is considered the major pathogen in the starting point and development of dental caries. *Streptococcus mutans* was also identified both in saliva and sub-gingival plaque samples in chronic periodontitis subjects and a positive correlation between *S. mutans* and the periodontal parameters was also detected^49^.

From the moment that CAP has emerged as a new tool to counteract bacterial biofilms, this study investigates CAP ability in removing biofilms developed by both *S. mutans* reference strain and microorganisms isolated from saliva of a volunteer with periodontitis as a representative model of biofilm in a periodontitis patient. The time points of CAP application (30,60,120 and 180 s) were chosen based on a possible CAP feasibility in a clinical situation.

A sufficient biofilm reduction is necessary for successful therapy of periodontal diseases^50^ and for this reason, three different analyses were made to quantify the effect of CAP on bacterial biofilms, as mentioned above. CAP was able to reduce both *S. mutans* and the saliva biofilm in a time-dependent manner. In detail, after 60 s of treatment both biofilms were reduced and after 120 s of treatment the number of viable cells were intensely lowered, as demonstrated by CFU count and XTT assay. CLSM analysis also showed that CAP was able to kill the bacteria within the biofilms, given that almost all the cells were yellow–red. Different results were observed when analysing the biofilm biomass after CAP treatment. The CV assay showed that the biofilm biomass persisted inside the wells, after CAP treatment at all time points, indicating that CAP alone was not able to remove the biofilm EPS matrix. These results are in accordance with those obtained by Jungbauer and co-authors, who found no correlation between the biofilm cells viability and the biofilm biomass of multispecies oral biofilm treated with an air CAP device^51^. Furthermore, the results of the CV assay are also corroborated by the CLSM results. CLSM images of *S. mutans* biofilm showed that the cells within the biofilms were dead after CAP treatment whereas the biomass continued to be evident. Regarding the saliva biofilm, CLSM images illustrated that CAP was able in reducing the biofilm thickness after 120 s and 180 s of treatment. It can be hypothesized that CAP treatment produces reactive species which kills the bacterial cells and only reduces the biofilm biomass, without totally removing it. The differences in CAP effectiveness in removing the biomass of *S. mutans* and saliva biofilms can be explained by the different media used to develop the two biofilms: for *S. mutans*, BHI plus 1% of sucrose was used whereas for the saliva biofilm only BHI was used. The adjunct of sucrose allows *S. mutans* to better adhere to a substrate and to form a strong biofilm.

The results obtained in our study are consistent with data obtained by other air plasma devices used against bacterial biofilms. In literature only a few publications related to the study of the effects of air-generated plasma against oral biofilms are available. Liguori and co-authors exposed *S. mutans* and *Aggregatibacter actinomycetemcomitans* single-species biofilm with two DBD plasma sources for different time points and analysed the decontamination effect via the CFU count and the XTT assay. Plasma generated from air induced a 2Log_10_ reduction in the CFU count and 20–40% loss in bacterial viability^52^. Cold air plasmas of DC and pulsed corona discharges were applied for 2, 5 and 10 m on *Streptococci* biofilms developed on plastic surfaces to assess the bio-decontamination effect. A reduction of bacterial population increased with the exposure time, reaching up to 2.5 log_10_ after 10 m of treatment, with no significant differences between positive and negative DC corona discharges^53^. A mature multilayer *Enterococcus faecalis* biofilm was treated with room-temperature, battery operated, handheld air plasma jet for 5 m at a fixed distance of 5 mm. The reactive plasma species produced by the plasma jet were able to penetrate to the bottom layer of the 25.5 μm-thick *E. faecalis* biofilm and to produce a strong bactericidal effect as demonstrated by the CLSM images showing that almost all cells were dead^54^. Hui and co-authors applied their air CAP device on a multispecies biofilms formed starting from the saliva of a peri-implantitis patient^55,56^. The authors developed an air plasma treatment by using a spark plasmapen, keeping a distance of 5 mm from the plasma nozzle tip to titanium discs surface, on which the saliva biofilm was formed. The biofilm removal, induced by the CAP device and demonstrated via the CV assay and SEM analysis, was comparable to the biofilm removal obtained by the air abrasion treatment. Indeed, plasma treatment was able to degrade the oral biofilms formed in situ on titanium discs without causing any thermal or mechanical damage on these surfaces.

It should be advantageous the application of CAP in the oral cavity without undesired side effects on gingival cells. In our study the cytotoxicity test carried out by the MTS assay showed that the average viability of hGF cells did not decrease below 50% until 120 s of exposure to plasma. CAP treatment for 30 s and 60 s did not significantly affect the viability and the morphology of hGF cells after direct application, even if a moderate cytotoxicity has been shown after 30 s and 60 s of CAP treatment. These data agree with the data obtained by Lee and co-authors who detected no cytotoxicity for hGF cells after air CAP treatment for 1 m^57^. Regarding our study, after 120 s of treatment the viability of hGF decreased until a maximum of 50% following cell morphology chaning. These results are similar to data obtained from another air plasma device used against bacterial biofilms: hGF cell viability did not decrease below 50% up to 150 s of plasma exposure^57^. Consequently, for a possible clinical application, is desirable applying this air CAP device for maximum 60 s which is the time point which resulted in a bactericidal effect without affecting hGF cell viability.

In conclusion, the CAP treatment can significantly reduce preformed in vitro biofilms developed by both *S. mutans* UA159 and the saliva microorganisms isolated by a donor affected by periodontitis. Further studies should be conducted on biofilms developed by additional saliva donors to support the potential of this innovative strategy to counteract oral pathogens responsible for periodontal diseases. From the moment that CAP has different mechanisms of action than currently used antimicrobial drugs, a future possible application in clinical field as a new tool to counteract antimicrobial resistance could be speculated.

## Methods

### Plasma system setup, plasma diagnosis, and ROS-RNS detection approach

The schematic structure of the air CAP employed in this study is presented in Fig. 1A. To generate the plasma, atmospheric air was used along with various components, such as a high voltage electrode, a ground electrode, a high-voltage power supply, and dielectrics. The feed air gas flow rate was maintained at a constant 2 L per minute (l pm), and a voltage controller was utilized to regulate the primary voltage. A conventional injection needle made of stainless-steel measuring 1.20 mm by 0.27 mm was used as the inner electrode for the high voltage electrode. In addition, stainless steel makes up the outside electrode that served as the ground electrode. The outer electrode had a thickness of 0.27 mm, a length of 6.00 mm, and included a centrally perforated hole measuring 0.70 mm to facilitate plasma generation. The discharge gap distance between the inner and outer electrodes was set at 2 mm. A gap distance of 6 mm was maintained between the tip of the plasma jet and the surface of the biofilm.

A digital oscilloscope (WaveSurfer 434, LeCroy, New York, NY, USA) was used to measure the peak voltage and current, along with a high-voltage probe (P6015A, Tektronix, Beaverton, OR, USA) and a current probe (CP030, LeCroy, Chestnut Ridge, NY, USA) attached to it. Additionally, optical emission spectra (OES) were recorded and ROS and RNS produced by the air cold plasma soft jet (air CAP) were analyzed using a fiber optic spectrometer (HR4000, Ocean Optics, Orlando, FL, USA). By employing the Modified Nitrogen collisional radiative model, the electron temperature (kT_e_), electron density (N_e_), and densities of nitrogen meta-stable states (n_A_, n_B_, and n_C_) were determined^58^. Furthermore, the vibrational temperature (T_v_) and rotational temperature (T_g_) of the plasma were calculated using a Boltzmann plot, utilizing the nitrogen second positive system^59^. Real-time gas-phase Fourier transform infrared spectroscopy (FTIR) was performed on the plasma using the MATRIX-MG5 automated FTIR gas analyzer and OPUS GA software. To conduct the analysis, the gas cell of the analyzer, equipped with a heating system, was maintained at a temperature of 25 °C throughout the study. The gas outlet of the air CAP system was connected to the gas analyzer to enable the continuous measurement of the plasma composition in real time. The conductivity and oxidation–reduction potential of the plasma-treated deionized water (DW) with volume of 500 μL were determined using a CON^30^ Tester and an ORP^30^ (Clean Instruments, Shanghai, China) tester, respectively.

### Saliva collection

A systemic healthy volunteer with no evidence of active caries, no salivary gland disease, no taking any drugs but affected by periodontitis was selected to donate his saliva. The approval the IRB was deemed unnecessary according to the regulations of region Abruzzo ethical Committee (protocol number: 0310899/23). The patient signed a written informed consent and the principles outlined in the declaration of Helsinki on clinical research involving human subjects were adhered to. To avoid diet interferences and circadian variations, the collection was performed between 8 and 11 am. The patient was asked to avoid eating or drinking any beverages (with exception of still water), taking drugs and to refrain from practicing oral hygiene routine at least for 12 h before saliva collection in the morning. The healthy volunteer was asked to rinse the mouth with ultrapure water and to spit from the bottom of the oral cavity into plastic calibrated containers (50 mL Falcon tube, Falcon, Corning, NY, USA). Ten milliliters of saliva were collected and immediately processed. In detail, the saliva sample was centrifuged at 4 °C at 3200×*g* for 20 m. The supernatant was discarded, and the pellet was resuspended in 10 mL of Brain Heart Infusion Broth (BHIB; Oxoid Limited, Hampshire, UK) and subsequently stored at − 80 °C in 15% glycerol stock.

### *Biofilm formation of the reference strain Streptococcus mutans* UA 159

*Streptococcus mutans* UA 159, a well characterized strain used in recent studies^60,61^, was used in this study. The bacterium was stored at − 80 °C before being thawed at room temperature, plated on Tryptone Soya Agar (TSA; Oxoid Limited, Basingstoke, Hampshire, UK) with 5% (v/v) of defibrinated horse sterile blood (Oxoid Ltd.), for 24 h, with 5% of CO_2_. Subsequently, a bacterial inoculum was prepared by picking colonies of *S. mutans* grown over the agar plate, by suspending them in BHIB, and by incubating for 17 h, at 125 rpm, at 37 °C with 5% of CO_2_. After the incubation, the biofilm was developed on 48-well flat-bottomed polystyrene microtiter plates (Falcon, Corning, NY, USA). In more detail, the OD_600_ of the overnight inoculum was read and adjusted to reach a final concentration in each well of 1.0 × 10^5^ CFU/mL. *Streptococcus mutans* biofilm was developed in BHIB + 1% of sucrose for 24 h of incubation at 37 °C, in static conditions with 5% of CO_2_, as previously reported^60^.

### Evaluation of biofilm formation from microorganisms isolated from a saliva sample after 24, 48 and 72 h of incubation

Saliva microorganisms stored at − 80 °C, as previously described, were plated onto TSA plus 5% of blood and incubated at 37 °C in anaerobic conditions for 48 h. Subsequently, a bacterial inoculum was prepared starting from the saliva microorganisms grown over the agar plate and by suspending them in BHIB. After 18 h of incubation in anaerobiosis, at 125 rpm, the OD_600_ was read (Spark® multimode microplate reader, Tecan Trading AG, Switzerland), and adjusted to reach a final concentration in each well of 1.0 × 10^7^ CFU/mL; 100 µl of the adjusted inoculum were used for forming the biofilm and the plates were incubated for 24, 48 and 72 h at 37 °C in anaerobic conditions. The biofilm of the microorganisms isolated from the saliva sample was developed on 96-well flat-bottomed polystyrene microtiter plates (Falcon, Corning, NY, USA), in BHIB. The biofilm formation was measured by using three different methods such as Colony Forming Units (CFU) counting, CV assay (Sigma Aldrich, St. Louis, MO, USA) and XTT metabolic assay (sodium 3´-[1-(phenylaminocarbonyl)-3,4- tetrazolium]-bis-(4-methoxy-6-nitro) benzene sulfonic acid hydrate, Cell Proliferation Kit II (XTT, Roche Diagnostic, Mannheim, Germany), according to the manufacturer’s recommendations, as previously described^60^.

### Confocal laser scanning microscopy (CLSM) analysis

CLSM analysis was carried out to evaluate the biofilm structure and thickness. The biofilms formed in 35 mm petri dishes (Ibidi GmbH, Planegg, Germany) were stained with Live/Dead™ BacLight™ Bacterial Viability Kit (Life Technologies Carlsbad, CA, USA) as previously reported^62^. Images were randomly taken for each experimental condition via a motorized table SMC 2009 and a multiple single position acquisition function (Tiles-Advanced setup, carrier 35 mm petri dish) using a confocal system Zeiss LSM800 (LSM 800, Zeiss, Germany) equipped with an inverted microscope Axio-observer.Z1 and a Plan-Apochromat 63 × /1.4 oil DIC objective and additional 1.5 × magnification. Biofilm structure and thickness were analyzed by acquiring a series of horizontal sections (stacks), with a thickness of 1.00 μm over the entire biofilm. The number of the optical planes were in the range of 8–33, chosen based on the size of treated and untreated biofilms. All images were acquired at a resolution of 1024 × 1024 pixels at 16 bit and processed via Zen Blue software (Carl Zeiss, Germany). Acquisition settings (laser power, photomultiplier gain, pin-hole and offset) were kept constant between different acquisition sessions.

### Sample and DNA extraction

The pellets used for the DNA extraction were the planktonic cells, derived from the overnight broth culture of saliva microorganisms, which were used to form the biofilm at each time point and the biofilm cells collected after 24, 48, and 72 h of incubation. In detail, the biofilm was obtained in 35 mm petri dishes, as described above. The planktonic cells were centrifuged at 3200×*g* at 4 °C for 20 m, were washed with Phosphate Buffered Saline (PBS; Sigma Aldrich, St. Louis, MO, USA) and centrifuged again in the same conditions. Subsequently, the biofilm was scraped and washed in PBS; the biofilm cells were removed by a scraper and collected by centrifugation at 3200×*g* at 4 °C for 20 m. After removing the washing PBS, the pellets of the biofilm formed at each time point as well as the pellets derived from the planktonic cells were used for the DNA extraction. All condition experiments were performed in triplicate.

Total DNA was extracted from pellet samples using the DNeasy PowerSoil Pro Kit (QIAGEN, Hilden, Germany). DNA concentrations were estimated by spectrophotometry using a NanoPhotometer® N60 (IMPLEN, Westlake Village, CA, USA), and the final concentration of the DNA sample was adjusted to 10 ng/µL. The quality of the purified DNA was assessed by agarose gels.

### 16S (V3–V4) region amplification, sequencing, and analysis

Samples for microbial community structure analysis based on the 16S (V3–V4) hypervariable region of DNA were amplified with universal primers^63^. Library preparation 16S ribosomal DNA (rDNA) gene sequencing and composition analysis of the bacterial colonies were performed by an external laboratory (BMR Genomics, Padova, Italy). Briefly, the variable V3 and V4 regions were amplified with universal prokaryote primers Pro341F and Pro805R. A Nextera XT Index kit (Illumina, San Diego, CA, USA) was used for libraries’ preparation. The library was created manually according to the manufacturer’s protocol. NGS was performed using the MiSeq instrument (Illumina, San Diego, CA, USA), and sequencing was performed with a 2 × 300-bp paired-end cycle.

Analyses of sequence reads were performed manually using the SILVA 16S 138 Database, which is available from the ARB website (https://www.arb-silva.de/). Reads obtained in the FastQ format were assigned to class levels with an 80% confidence threshold.

The raw sequences were verified and filtered by quality, trimmed by the primers, and fused with Quantitative Insights into Microbial Ecology 2 (Qiime2) bioinformatics platform, version qiime2 2021.4.0 software. Primers were removed using the Cutadapt plugin. Paired-end sequences were subjected to quality control including read quality filtering and trimming, error rate estimation, dereplication, read merging, and chimera detection using the DADA2 plugin implemented in QIIME 2. The resulting amplicon sequence variants (ASV) table was filtered at 0.001% to remove singletons and rare ASVs. Taxonomic classification of ASVs was carried out with the q2-feature-classifier plugin using trained operational taxonomic units (OTUs) at 99% from Silva database version 138. Samples were rarefied to sequences by random subsampling in QIIME2 before downstream alpha and beta diversity analyses. No samples were excluded by the rarefaction step. Metagenomic sequences are available at the National Centre for Biotechnology Information Sequence Read Archive (https://www.ncbi.nlm.nih.gov/sra/), BioProject number PRJNA1040003.

### Minimum biofilm eradication concentration (MBEC) of CAP source

Both biofilms were developed on 48-well flat-bottomed polystyrene microtiter plates by choosing the time of incubation of 72 h for the saliva biofilm and 24 h for *S. mutans* biofilm*.* CAP effect in reducing bacterial biofilms was measured by using CFU counting, XTT metabolic assay and CV assay. At the end of incubation, non-adherent cells were removed, and the biofilms were washed with PBS. The biofilms were then treated with the CAP source at a distance of 6 mm at different time points, corresponding to 30 s, 60 s, 120 s and 180 s. Immediately after CAP treatment, XTT was added into the wells and incubated at 37 °C for two hours in the appropriate conditions.

The absorbance, corresponding to 490 nm for XTT, was then read by using a microplate reader (Spark® multimode microplate reader, Tecan Trading AG, Switzerland). The Minimum Biofilm Eradication Concentration (MBEC) was defined as the lowest concentration of the sample resulting in a detectable colorimetric change of the wells. The colorimetric change is related to the metabolic activity of the cells inside a biofilm^60^.

CV assay was carried out to study the effect of CAP against the biofilm biomass following the protocol described by Vitale and co-authors , 2023^60^. Briefly, after the washing, both biofilms were dried at 60 °C for 1 h and 0.1% crystal violet was added for 2 min. Biofilms stained with CV were decolored with 33% acid acetic and absorbance was read at 590 nm. CAP effectiveness was expressed as percentage of the Optical Density measured in the wells that did not receive treatment (the control biofilm). Three independent experiments were performed in triplicate.

### Cell viability evaluation via colony-forming unit count

CFU count was performed to evaluate the bacterial viable count after CAP treatment. Treated and non-treated biofilms were taken from each well by scraping and used for the CFU counts. Serial dilutions of the stock were performed in PBS and plated on TSA plus 5% of blood at 37 °C for 48 h in the appropriate conditions.

### Cell lines, treatments and proliferation assay

hGF cells (human Gingival Fibroblasts cell line) (CLS Cell Lines Service GmbH, Germany) were routinely grown in Dulbecco’s Modified Eagle’s Medium (DMEM-F12) (EuroClone S.p.A., Pero (MI), Italy), supplemented with 5% fetal bovine serum (FBS) (Euroclone), 2 mM l-glutamine (Euroclone), 1% penicillin–streptomycin (Euroclone) and 10 mM HEPES.

For CAP direct treatments, hGF cells were seeded and cultured under an atmosphere of 95% air/5% CO_2_ at 37 °C overnight before treatment with CAP. The working distance between the capillary of the plasma device and the liquid medium surface was fixed at 6 mm.

Cell viability was determined by MTS [3-(4,5-dimethylthiazol-2-yl)-5-(3-carboxymethoxyphenyl)-2-(4-sulfophenyl)-2H-tetrazolium] assay (Promega, Madison WI, USA). hGF cell cultures, 24 h after seeding at a density of 15 × 10^3^ per well in 48 well-plates with 0.5 mL complete medium, were subjected to plasma direct treatment for 30 s, 60 s, 120 s and 180 s. After 24 h and 48 h, MTS solution (10% of the total medium volume) was added to each well and following incubation for one hour at 37 °C, the cell viability was evaluated by measuring the colorimetric absorbance at 490 nm using microplate reader (Spark® multimode microplate reader, Tecan Trading AG, Switzerland). The effect was expressed as percentage of the Optical Density measured in cultures that did not receive treatment (100% viability). Determinations were carried out in triplicate in three independent experiments.

### Cell morphology

hGF cells were cultured in 24-well culture plates (30 × 10^3^) containing round glass inserts and after 24 h, adherent cells were treated with plasma for 30 s, 60 s, 120 s and 180 s. Then they were incubated for 24 h and stained with 1% Toluidine Blue Solution (TAAB Laboratories Equipment Ltd, UK). Digital Images of three different experiments were obtained using microscopy connected with a camera at 40x (Leica, Wild Heer-brugg, Wetzlar, Germany).

### Statistical analysis

All the data are reported as the mean values of three independent experiments. In detail, data are expressed as the means ± SD. Regarding the biofilms assays, the differences in the means of the results between untreated and treated biofilms were evaluated by one way ANOVA (GraphPad Software, San Diego, CA, USA) and Dunnet’s multiple comparison test. Untreated biofilms grown in their medium were used as control. Regarding hGF cells, the data are analyzed by unpaired t test (two-tailed *p* value). A value of *p* < 0.05 was considered to indicate a statistically significant difference.

### Supplementary Information

Supplementary Figures.

## Data Availability

All datasets generated during the current study are available in the manuscript or from the corresponding authors upon request. Metagenomic sequences are available at the National Centre for Biotechnology Information Sequence Read Archive (https://www.ncbi.nlm.nih.gov/bioproject/?term=PRJNA1040003) with accession number PRJNA1040003.

## References

[1] Jorth P (2014). Metatranscriptomics of the human oral microbiome during health and disease. mBio.

[2] Chowdhry A, Kapoor P, Bhargava D, Bagga DK (2023). Exploring the oral microbiome: An updated multidisciplinary oral healthcare perspective. Discoveries.

[3] Flemming HC (2016). Biofilms: An emergent form of bacterial life. Nat. Rev. Microbiol..

[4] Grande, R., Puca, V. & Muraro, R. Antibiotic resistance and bacterial biofilm. **30**, 897–900. 10.1080/13543776.2020.1830060 (2020) 10.1080/13543776.2020.1830060 32985275

[5] Cooper, R. A., Bjarnsholt, T. & Alhede, M. Biofilms in wounds: A review of present knowledge, **23**, 570–582. 10.12968/jowc.2014.23.11.570 (2014). 10.12968/jowc.2014.23.11.570 25375405

[6] Lamont RJ, Koo H, Hajishengallis G (2018). The oral microbiota: Dynamic communities and host interactions. Nat. Rev. Microbiol..

[7] Sedghi L, DiMassa V, Harrington A, Lynch SV, Kapila YL (2021). The oral microbiome: Role of key organisms and complex networks in oral health and disease. Periodontology.

[8] Yan J, Bassler BL (2019). Surviving as a community: Antibiotic tolerance and persistence in bacterial biofilms. Cell Host Microbe.

[9] Townsend EM (2016). Development and characterisation of a novel three-dimensional inter-kingdom wound biofilm model. Biofouling.

[10] Ten threats to global health in 2019. https://www.who.int/news-room/spotlight/ten-threats-to-global-health-in-2019.

[11] Murray CJ (2022). Global burden of bacterial antimicrobial resistance in 2019: A systematic analysis. The Lancet.

[12] Jepsen K (2021). Prevalence and antibiotic susceptibility trends of periodontal pathogens in the subgingival microbiota of German periodontitis patients: A retrospective surveillance study. J. Clin. Periodontol..

[13] Shaw P (2018). Bacterial inactivation by plasma treated water enhanced by reactive nitrogen species. Sci. Rep..

[14] Shaw P (2019). Synergistic effects of melittin and plasma treatment: A promising approach for cancer therapy. Cancers.

[15] Shaw P, Kumar N, Privat-maldonado A, Smits E, Bogaerts A (2021). Cold atmospheric plasma increases temozolomide sensitivity of three-dimensional glioblastoma spheroids via oxidative stress-mediated dna damage. Cancers (Basel).

[16] Kumar N (2021). Physical plasma-derived oxidants sensitize pancreatic cancer cells to ferroptotic cell death. Free Radic. Biol. Med..

[17] Kumar N (2016). The action of microsecond-pulsed plasma-activated media on the inactivation of human lung cancer cells. J. Phys. D Appl. Phys..

[18] Kumar N (2018). Enhancement of cellular glucose uptake by reactive species: A promising approach for diabetes therapy. RSC Adv..

[19] Kumar N, Attri P, Dewilde S, Bogaerts A (2018). Inactivation of human pancreatic ductal adenocarcinoma with atmospheric plasma treated media and water: A comparative study. J. Phys. D Appl. Phys..

[20] Jha N, Ryu JJ, Choi EH, Kaushik NK (2017). Generation and role of reactive oxygen and nitrogen species induced by plasma, lasers, chemical agents, and other systems in dentistry. Oxidative Med. Cell. Longev..

[21] Yusupov M (2021). Oxidative damage to hyaluronan–CD44 interactions as an underlying mechanism of action of oxidative stress-inducing cancer therapy. Redox Biol..

[22] Privat-Maldonado A (2019). ROS from physical plasmas: Redox chemistry for biomedical therapy. Oxidative Med. Cell. Longev..

[23] Morfill GE, Kong MG, Zimmermann JL (2009). Focus on plasma medicine. New J. Phys..

[24] Yan D (2016). Stabilizing the cold plasma-stimulated medium by regulating medium’s composition. Sci. Rep..

[25] Ghimire B (2019). The role of UV photolysis and molecular transport in the generation of reactive species in a tissue model with a cold atmospheric pressure plasma jet. Appl. Phys. Lett..

[26] Laroussi M (2014). From killing bacteria to destroying cancer cells: 20 Years of plasma medicine. Plasma Process. Polym..

[27] Schmidt A, von Woedtke T, Vollmar B, Hasse S, Bekeschus S (2019). Nrf2 signaling and inflammation are key events in physical plasma-spurred wound healing. Theranostics.

[28] Chawla K, Lamba A, Tandon S, Faraz F, Gaba V (2016). Effect of low-level laser therapy on wound healing after depigmentation procedure: A clinical study. J. Indian Soc. Periodontol..

[29] Ahn HJ (2014). Targeting cancer cells with reactive oxygen and nitrogen species generated by atmospheric-pressure air plasma. PLoS One.

[30] Chen Z, Cheng X, Lin L, Keidar M (2016). Cold atmospheric plasma discharged in water and its potential use in cancer therapy. J. Phys. D Appl. Phys..

[31] Almeida ND (2019). Cold atmospheric plasma as an adjunct to immunotherapy for glioblastoma multiforme. World Neurosurg..

[32] Van Loenhout J (2021). Auranofin and cold atmospheric plasma synergize to trigger distinct cell death mechanisms and immunogenic responses in glioblastoma. Cells.

[33] Filipić A, Gutierrez-Aguirre I, Primc G, Mozetič M, Dobnik D (2020). Cold plasma, a new hope in the field of virus inactivation. Trends Biotechnol..

[34] Wang G (2016). Non-thermal plasma for inactivated-vaccine preparation. Vaccine.

[35] Sklias K, Sousa JS, Girard PM (2021). Role of short- and long-lived reactive species on the selectivity and anti-cancer action of plasma treatment in vitro. Cancers.

[36] Bernhardt T (2019). Plasma medicine: Applications of cold atmospheric pressure plasma in dermatology. Oxidative Med. Cell. Longev..

[37] Krewing M (2019). Plasma-sensitive *Escherichia coli* mutants reveal plasma resistance mechanisms. J. R. Soc. Interface.

[38] Mai-Prochnow A, Bradbury M, Ostrikov K, Murphy AB (2015). *Pseudomonas aeruginosa* biofilm response and resistance to cold atmospheric pressure plasma is linked to the redox-active molecule phenazine. PLoS One.

[39] Lamichhane P (2022). Non-thermal argon plasma jets of various lengths for selective reactive oxygen and nitrogen species production. SSRN Electron. J..

[40] Acharya TR, Lee GJ, Choi EH (2022). Influences of plasma plume length on structural, optical and dye degradation properties of citrate-stabilized silver nanoparticles synthesized by plasma-assisted reduction. Nanomaterials.

[41] Dall GF, Tsang SJ, Gwynne PJ (2018). Unexpected synergistic and antagonistic antibiotic activity against Staphylococcus biofilms. J. Antimicrob. Chemother..

[42] Hosseinpour-Nader A, Karimi N, Ghafari HA (2023). Ex-vivo effects of propolis quantum dots-nisin-nanoquercetin-mediated photodynamic therapy on *Streptococcus mutans* biofilms and white spot lesions. Photodiagn. Photodyn. Ther..

[43] Attri P (2015). Influence of reactive species on the modification of biomolecules generated from the soft plasma. Sci. Rep..

[44] Lamichhane P (2023). Surface activation of thin polyvinyl alcohol films by atmospheric pressure plasma jet: Influence of electron temperature. Plasma Process. Polym..

[45] Dhakal OB (2023). Effects of spark dielectric barrier discharge plasma on water sterilization and seed germination. CAP.

[46] HOMD: Human Oral Microbiome Database. https://www.homd.org/.

[47] Dewhirst FE (2010). The human oral microbiome. J. Bacteriol..

[48] Verma D, Garg PK, Dubey AK (2018). Insights into the human oral microbiome. Arch. Microbiol..

[49] Dani S (2016). Assessment of *Streptococcus mutans* in healthy versus gingivitis and chronic periodontitis: A clinico-microbiological study. Contemp. Clin. Dent..

[50] Sanz M (2020). Treatment of stage I-III periodontitis-The EFP S3 level clinical practice guideline. J. Clin. Periodontol..

[51] Jungbauer G, Favaro L, Müller S, Sculean A, Eick S (2022). The in-vitro activity of a cold atmospheric plasma device utilizing ambient air against bacteria and biofilms associated with periodontal or peri-implant diseases. Antibiotics.

[52] Liguori A (2017). Cold atmospheric plasma treatment affects early bacterial adhesion and decontamination of soft reline palatal obturators. Clin. Plasma Med..

[53] Kovalová Z, Tarabová K, Hensel K, MacHala Z (2013). Decontamination of Streptococci biofilms and Bacillus cereus spores on plastic surfaces with DC and pulsed corona discharges. Eur. Phys. J. Appl. Phys..

[54] Pei X (2012). Inactivation of a 25.5 µm *Enterococcus faecalis* biofilm by a room-temperature, battery-operated, handheld air plasma jet. J. Phys. D Appl. Phys..

[55] Hui WL (2021). Novel technique using cold atmospheric plasma coupled with air-polishing for the treatment of titanium discs grown with biofilm: An in-vitro study. Dent. Mater..

[56] Hui WL (2021). Cold atmospheric plasma coupled with air abrasion in liquid medium for the treatment of peri-implantitis model grown with a complex human biofilm: An in vitro study. Clin. Oral Investig..

[57] Lee JH (2016). Selective killing effects of cold atmospheric pressure plasma with NO induced dysfunction of epidermal growth factor receptor in oral squamous cell carcinoma. PLoS One.

[58] Matthes R (2013). Antimicrobial efficacy of two surface barrier discharges with air plasma against in vitro biofilms. PLoS One.

[59] Choi EH, Uhm HS, Kaushik NK (2021). Plasma bioscience and its application to medicine. AAPPS Bull..

[60] Vitale I (2023). Antibiofilm activity and NMR-based metabolomic characterization of cell-free supernatant of Limosilactobacillus reuteri DSM 17938. Front. Microbiol..

[61] Palmer SR (2019). *Streptococcus mutans* yidC1 and yidC2 impact cell envelope biogenesis, the biofilm matrix, and biofilm biophysical properties. J. Bacteriol..

[62] Grande R (2021). Selective inhibition of helicobacter pylori carbonic anhydrases by carvacrol and thymol could impair biofilm production and the release of outer membrane vesicles. Int. J. Mol. Sci..

[63] Takahashi S, Tomita J, Nishioka K, Hisada T, Nishijima M (2014). Development of a prokaryotic universal primer for simultaneous analysis of *Bacteria* and *Archaea* using next-generation sequencing. PLoS One.

