# Supplementary material for: Antimicrobial efficacy of direct air gas soft jet plasma for the in vitro reduction of oral bacterial biofilms
Source: Sci Rep. 2024 May 13;14:10882. doi: 10.1038/s41598-024-61438-z (PMC11091221; doi:10.1038/s41598-024-61438-z)
Supplement: Supplementary file 1 — Supplementary Figures. [file 41598_2024_61438_MOESM1_ESM.docx]

**Supplementary information**

**Title: Antimicrobial Efficacy of Direct Air Gas Soft Jet Plasma for the *in Vitro* Reduction of Oral Bacterial Biofilms**

**Authors:** Valentina Puca^1^, Beatrice Marinacci^1,2^, Morena Pinti^1^, Federica Di Cintio^3,6^, Bruna Sinjari^2^, Maria Carmela Di Marcantonio^2^, Gabriella Mincione^2^, Tirtha Raj Acharya^4^, Nagendra Kumar Kaushik^4^, Eun Ha Choi^4^, Michele Sallese^2,6^, Simone Guarnieri^5,6^, Rossella Grande^1,6^* and Vittoria Perrotti^2,7^*

^1^Department of Pharmacy, University "G. d'Annunzio", Chieti-Pescara, Chieti, Italy; valentina.puca@unich.it; beatrice.marinacci@unich.it; pintimorenavalentina@gmail.com; rossella.grande@unich.it;

^2^Department of Innovative Technologies in Medicine & Dentistry, University "G. d'Annunzio", Chieti-Pescara, Chieti; b.sinjari@unich.it; dimarcantonio@unich.it; gabriella.mincione@unich.it; michele.sallese@unich.it; [v.perrotti@unich.it](mailto:v.perrotti@unich.it)

^3^Department of Oral, Medical and Biotechnological Sciences, University "G. d'Annunzio", Chieti-Pescara, Chieti; federica.dicintio@unich.it;;

^4^Plasma Bioscience Research Center, Department of Electrical and Biological Physics, Kwangwoon University, Seoul 01897, South Korea; [tirtharajacharya2050@gmail.com](mailto:tirtharajacharya2050@gmail.com); kaushik.nagendra@kw.ac.kr; ehchoi@kw.ac.kr;

^5^Department of Neuroscience, Imaging and Clinical Sciences, University "G. d'Annunzio", Chieti-Pescara, Chieti; simone.guarnieri@unich.it;

^6^Center for Advanced Studies and Technology (CAST), University "G. d'Annunzio", Chieti-Pescara, Chieti.

^7^UdA-TechLab, Research Center, University “G. d’Annunzio” of Chieti-Pescara, 66100 Chieti, Italy;

* These two authors share senior authorship

**This PDF file includes:**

**-** Supplementary Figure S1

- Supplementary Figure S2

- Supplementary Figure S3


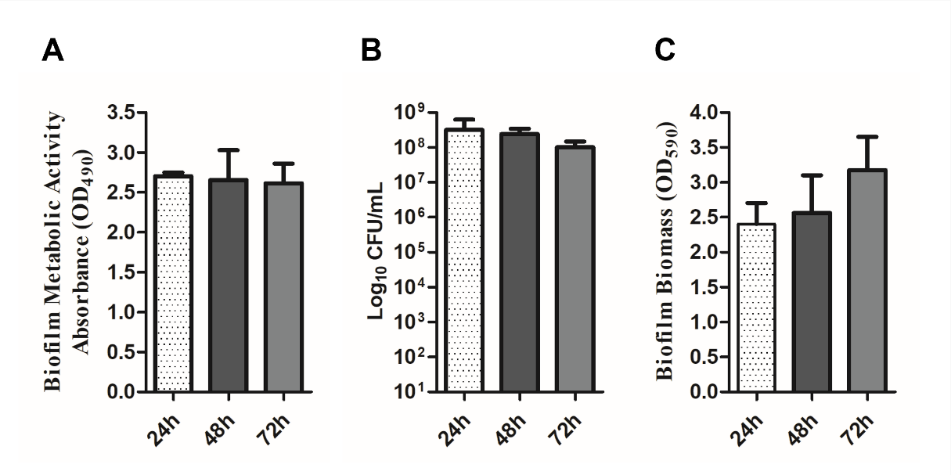


**Supplementary Figure S1.** Evaluation of the biofilm formed by the microorganisms isolated from a saliva sample after 24, 48 and 72h of incubation. biofilm metabolic activity absorbance (XTT assay) (A); CFU count(B); biofilm biomass measured via CV staining (C). The results represent the mean±SD of three independent experiments.


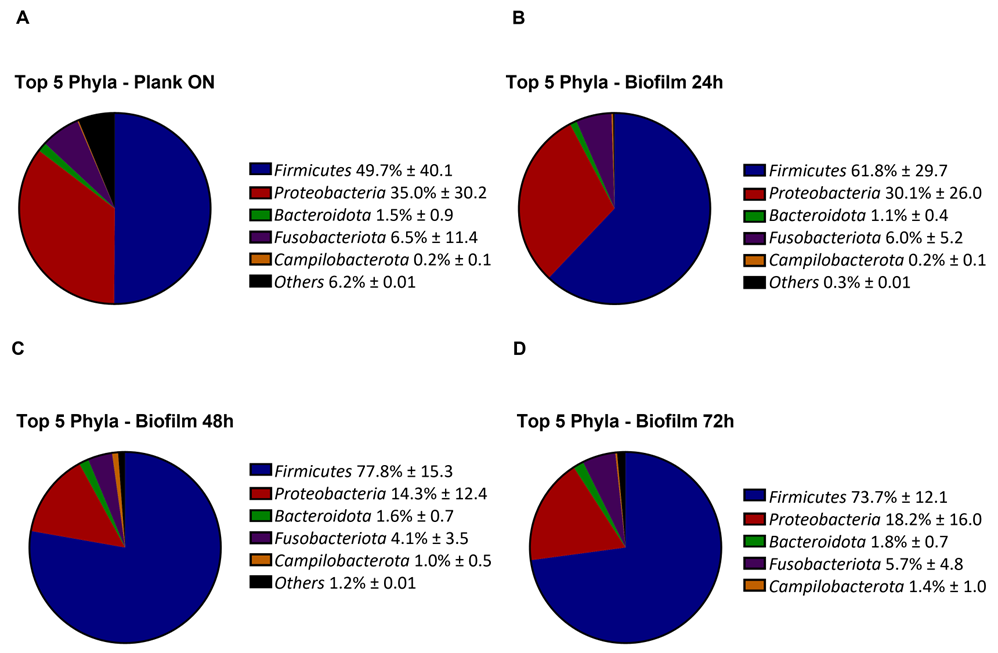


**Supplementary Figure S2.** Characterization of microbial fraction of planktonic and biofilm cells by *phyla*.

Comparison of *phyla* composition of planktonic ON (A) and biofilm after 24h (B), 48h (C), and 72h (D) of incubation. The top 5 *phyla* in terms of maximum relative abundance are shown. All other *phyla* with low presence (≤ 0.7) were grouped as “Other”. All Data are expressed as means ± SD (n=3).


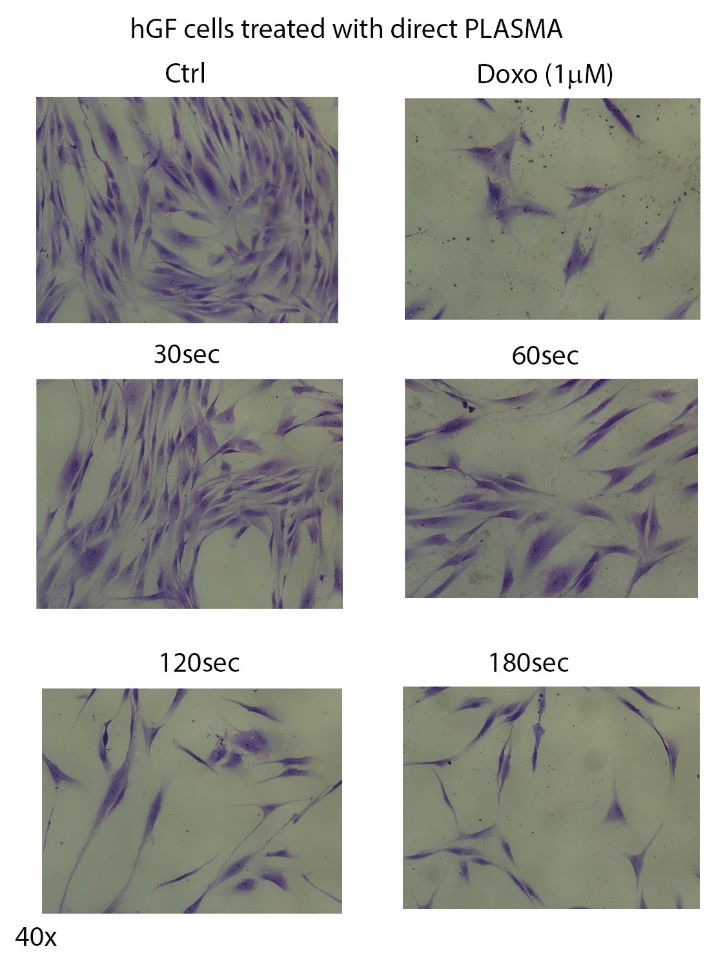


**Supplementary Figure S3.** Effects of direct CAP treatment on hGF cells morphology. CTRL: control, untreated cells; Doxo (1μM); CAP treatment for 30 sec, 30 seconds; 60sec, 60 seconds; 120sec, 120s seconds; 180sec, 180 seconds. Inverted optical microscopy images were acquired at a total magnification: 400x.
